# Recruitment of Brd3 and Brd4 to acetylated chromatin is essential for proinflammatory cytokine-induced matrix-degrading enzyme expression

**DOI:** 10.1186/s13018-019-1091-3

**Published:** 2019-02-20

**Authors:** Jin Dai, Sheng Zhou, Qiting Ge, Jinzhong Qin, Jianxin Li, Huangxian Ju, Yi Cao, Minghao Zheng, Chaojun Li, Xiang Gao, Huajian Teng, Qing Jiang

**Affiliations:** 1 The Center of Diagnosis and Treatment for Joint Disease, Drum Tower Hospital, Medical School, Nanjing University, Zhongshan Road 321, Nanjing, 210008 Jiangsu People’s Republic of China; 2 The Model Animal Research Center of Nanjing University, Xuefu Road, Nanjing, 210032 Jiangsu People’s Republic of China; 3 State Key Laboratory of Analytical Chemistry for Life Science, Nanjing University, Hankou Road, Nanjing, 210093 People’s Republic of China; 4 Collaborative Innovation Center of Advanced Microstructures, National Laboratory of Solid State Microstructure and Department of Physics, Nanjing University, Hankou Road, Nanjing, 210093 People’s Republic of China; 5 Sir Charles Gairdner Hospital, School of Surgery, The University of Western Australia, 35 Stirling Highway, Perth, 6009 Australia; 6 State Key Laboratory of Pharmaceutical Biotechnology and Jiangsu Key Laboratory of Molecular Medicine, Model Animal Research Center and School of Medicine, Nanjing University, Nanjing, 210093 People’s Republic of China

**Keywords:** Matrix-degrading enzymes, Brd, Transcription, Acetylation

## Abstract

**Background:**

Proinflammatory cytokines, which can upregulate the expression of matrix-degrading enzymes in chondrocytes, play important roles in the development of osteoarthritis. BET family proteins, acting as the “readers” of acetylated modifications on histones, have been linked to transcriptional regulation. And a BET protein inhibitor, I-BET151, has been shown to inhibit the induction of matrix-degrading enzymes by proinflammatory cytokines in chondrocytes. Our objective is to clarify the role and mechanism of BET proteins on matrix-degrading enzyme gene expression by using a human chondrosarcoma cell line (SW1353).

**Methods:**

We pretreated SW1353 cells with I-BET151 prior to treatment with IL-1β or TNF-α and then checked the expression of four matrix-degrading enzyme genes (MMP1, MMP3, MMP13, and ADAMTS4). We performed knockdown of BET protein family members (BRD2, BRD3, and BRD4) with corresponding siRNAs in SW1353 cells prior to treatment with IL-1β or TNF-α and checked the expression of the matrix-degrading enzyme genes. We evaluated Brd-mediated transcriptional regulation on the matrix-degrading enzyme genes by ChIP assay.

**Results:**

We confirmed that I-BET151 could suppress the IL-1β- or TNF-α-induced expression of *MMP1*, *MMP3*, *MMP13*, and *ADAMTS4* in SW1353 cells. Brd3 and Brd4 were required for the IL-1β- or TNF-α-induced expression of matrix-degrading enzyme genes in SW1353 cells. We revealed that inducible acetylation of H4k5/8/12 and the recruitment of Brd3, Brd4, and p-TEFb to chromatin were involved in IL-1β- or TNF-α-induced transcription.

**Conclusions:**

Our findings suggested that Brd3 and Brd4 were essential for the IL-1β- or TNF-α-induced transcription of matrix-degrading enzyme genes, and recruitment of Brd3 and Brd4 to chromatin of these genes played the main role in this process.

**Electronic supplementary material:**

The online version of this article (10.1186/s13018-019-1091-3) contains supplementary material, which is available to authorized users.

## Background

Osteoarthritis (OA) is a common multifactorial disorder of the joints and is mainly characterized by the progressive degeneration of articular cartilage. Matrix-degrading enzymes including matrix metalloproteinase (MMP) 1, MMP3, MMP13, a disintegrin and metalloproteinase with thrombospondin motifs (ADAMTS) 4, and ADAMTS5 played important roles in cartilage degradation in OA [1]. Among these matrix-degrading enzymes, MMP1, MMP3, MMP13, and ADAMTS4 can be upregulated by proinflammatory cytokines, such as interleukin (IL)-1β and tumor necrosis factor (TNF)-α. Signal transduction pathways, such as the nuclear factor-κB (NF-κB), Jun N-terminal kinase (JNK), and mitogen-activated protein kinase (MAPK) pathways, were involved in the process of activating matrix-degrading enzymes by proinflammatory cytokines [1–3].

Recently, a small molecular inhibitor which could block bromodomain and extraterminal domain (BET) proteins from recognizing acetylated histones (I-BET151) was generated [4], and we found I-BET151 could abrogate the induction of MMP1, MMP3, MMP13, and ADAMTS4 by IL-1β or TNF-α in chondrocytes [5]. In mammals, the BET family of proteins consists of ubiquitously expressed bromodomain-containing protein (Brd)2, Brd3, Brd4, and testes/oocyte-specific Brdt. All members of BET proteins contain two conserved tandem bromodomains capable of recognizing acetylated histones, which are involved in transcription regulation through the recruitment of transcriptional regulatory complexes to acetylated chromatin [6, 7]. There were studies which reported that IL-1β and TNF-α could increase the acetylation of histones within the promoter regions of different genes [8–10]. We suspected that enhanced recruitment of BET proteins to acetylated histones at the promoter regions was responsible for the induction of these matrix-degrading enzymes by IL-1β or TNF-α.

In the present study, we examined the role and mechanisms of BET proteins in the transcriptional regulation of matrix-degrading enzyme genes. We demonstrated that recruitment of BET proteins to acetylated chromatin is a crucial step for the induction of these matrix-degrading enzymes by IL-1β or TNF-α, and only Brd3 and Brd4 had a positive effect in this process.

## Methods

### Cell culture and treatment

Human chondrosarcoma cells (SW1353) were obtained from ATCC and cultured in Dulbecco’s Modified Eagle’s Medium (Invitrogen) supplemented with 10% fetal bovine serum (Invitrogen), 2 mM glutamine, 100 units/ml penicillin, and 100 g/ml streptomycin at 37 °C in an atmosphere of 5% CO_2_. Although SW1353 cell line has limitations for chondrocyte research, this cell line has been shown to be a suitable cell model for examining the regulation of many catabolic genes in chondrocytes [11], and high transfection efficiency can be obtained in this cell line. Human samples were obtained with informed consent from the donors. The study was approved by the ethical committee of Drum Tower Hospital, Medical School, Nanjing University. Fresh cartilage samples (from a patient who underwent knee replacement surgery at Drum Tower Hospital, Medical School, Nanjing University) were chopped from the lateral condyle of the operated knee. The patient was male and 65 years of age. He suffered with OA and had no history of immunological diseases. Primary human articular chondrocytes were cultured as previously described [12].

For the cellular assays, the cells were grown to approximately 80% confluence and then starved (in medium containing 0.1% FBS) for 12 h. I-BET151 (1 μM; TOCRIS, R&D Systems) or vehicle was added 1 h prior to treatment with IL-1β (10 ng/ml; R&D Systems) or TNF-α (10 ng/ml; R&D Systems). Dimethyl sulfoxide (DMSO) was used as the vehicle for I-BET151, IL-1β, and TNF-α.

### Gene expression analysis

The total RNA from primary human chondrocytes or SW1353 cells was isolated using TRIzol Reagent (Ambion, Invitrogen). First-strand cDNA was prepared by reverse transcription using the PrimeScript RT Reagent Kit according to the manufacturer’s manual (TaKaRa). Real-time PCR was performed in an ABI StepOnePlus instrument (Applied Biosystems) using SYBR Green PCR Master Mix (Thermo Scientific). Briefly, all real-time PCR was performed in a reaction volume of 20 μl, using 96-well optical grade PCR plates (Invitrogen). The reactions were conducted at 50 °C for 2 min, then at 95 °C for 10 min, and followed by 40 cycles of 95 °C for 15 s and at 60 °C for 60 s. The primers used in this study are listed in Additional file 1: Table S1. Each sample for real-time PCR was evaluated by three tests.

### RNA interference

Predesigned siRNAs specific to human *BRD2*, *BRD3*, and *BRD4*, as well as scrambled siRNA, were obtained from Santa Cruz Biotech (Santa Cruz Biotechnology). SW1353 cells were grown to approximately 70% confluency and were then treated with siRNA using Lipofectamine 2000 for 48 h prior to treatment with IL-1β or TNF-α.

### Western blot assay

Western blot assays were carried out as previously described [13]. The proteins were resolved on 8–12% SDS-polyacrylamide gels, as required, and transferred to BioTrace NT membranes (Pall, Life Sciences). The membranes were probed with the appropriate primary antibodies, and the proteins were detected using peroxidase-conjugated antibodies and visualized by ECL (Pierce, Thermo). The primary antibodies used were rabbit polyclonal anti-Brd4 (1:1000; Cell Signaling Technology), rabbit monoclonal anti-Brd2 (1:1000; Abcam), mouse monoclonal anti-Brd3 (1:500; Abcam), and anti-glyceraldehyde-3-phosphate dehydrogenase (GAPDH) (1:1000; Santa Cruz Biotechnology, INC).

### ChIP assay

ChIP assays were carried out using a commercially available kit according to the manufacturer’s instructions (Upstate, Millipore). Briefly, human chondrocytes were fixed, harvested, and sonicated. The sonicated supernatants were diluted with ChIP dilution buffer and precleared with protein G agarose. After removing 1% supernatants as input, the supernatants were incubated with an antibody against Brd3 (Abcam), Brd4 (Abcam), cyclin-dependent kinase 9 (Cdk9) (Santa Cruz Biotechnology, INC), Ser2-phosphorylated RNA polymerase II C-terminal domain (S2P Pol II) (Abcam), H4K5Ac (Millipore), H4K8Ac (Millipore), H4K12Ac (Millipore), and nonspecific IgG (Upstate, Millipore) antibodies overnight at 4 °C with rotation. The protein/DNA complexes and the input were eluted, reversed, and purified. The quantitative analysis of targeted promoter regions was determined by real-time PCR using specific primers (Additional file 1: Table S1), and the results were normalized to the level of input by using the same primers. Each sample for real-time PCR was evaluated by three tests.

### Statistical analysis

All data were expressed as the means SE and represent at least two independent experiments. Statistical comparisons were made using Student’s *t* test. *P* < 0.05 was considered statistically significant.

## Results

### Brd3 and Brd4 are required for expression of IL-1β- or TNF-α-induced matrix-degrading enzymes

**Fig. 1:**
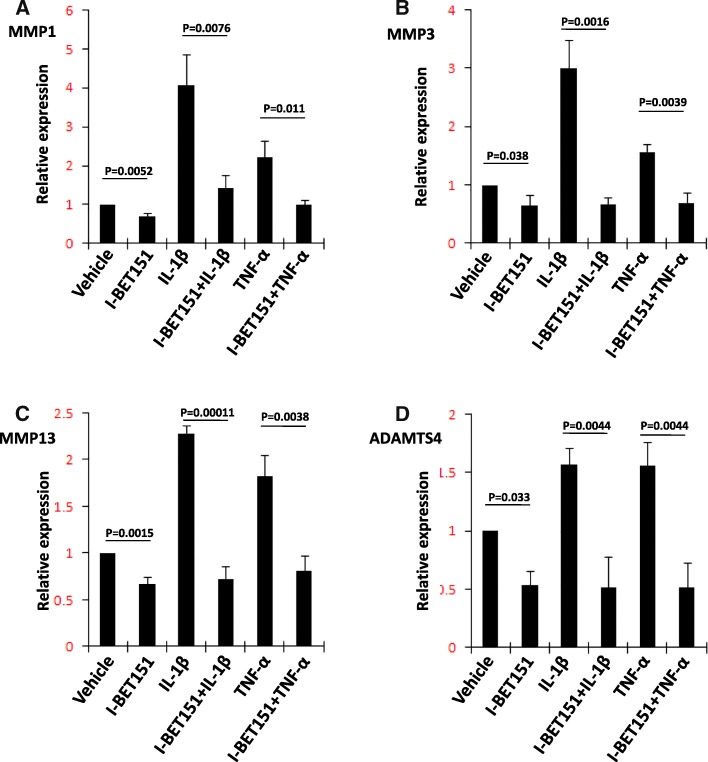
The effect of I-BET151 on regulation of matrix-degrading enzyme gene transcription in SW1353 cells. **a**–**d** The transcriptional expression (RT-PCR) of *MMP1*, *MMP3*, *MMP13*, and *ADAMTS4* genes in SW1353 cells, respectively, after the cells were pretreated with or without I-BET151 (1 μM) followed by addition of vehicle, IL-1β (10 ng/ml) or TNF-α (10 ng/ml) for 6 h. Relative fold-change values were calculated in comparison with vehicle control that was set to 1 (*n* = 3)

**Fig. 2:**
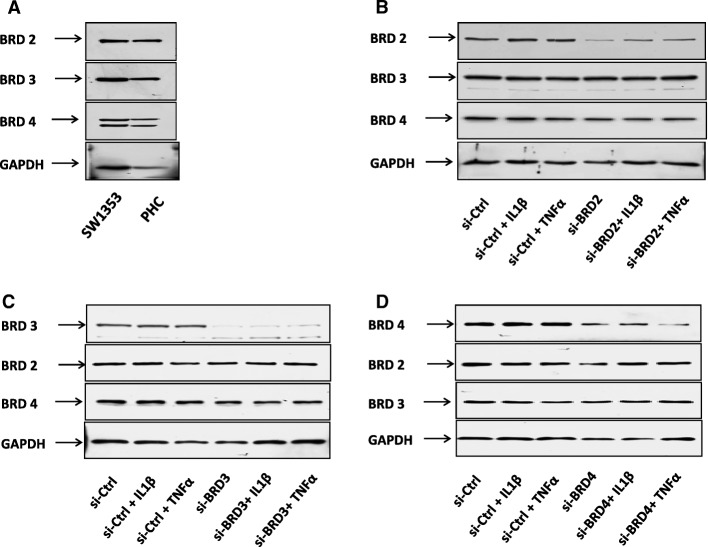
The protein expression of the BET family members and the effect of siRNAs in SW1353 cells. **a** The protein expression of Brd2, Brd3, and Brd4 was confirmed by Western blot assay in SW1353 cells and primary human chondrocytes, respectively. PHC, primary human chondrocyte. **b**–**d** SW1353 cells were treated with scrambled siRNA or specific siRNA targeting *BRD2*, *BRD3*, and *BRD4* for 48 h and then treated with or without IL-1β (10 ng/ml) or TNF-α (10 ng/ml) for 6 h. The presence of Brd2, Brd3, and Brd4 proteins in SW1353 cells was confirmed by Western blot assay

**Fig. 3:**
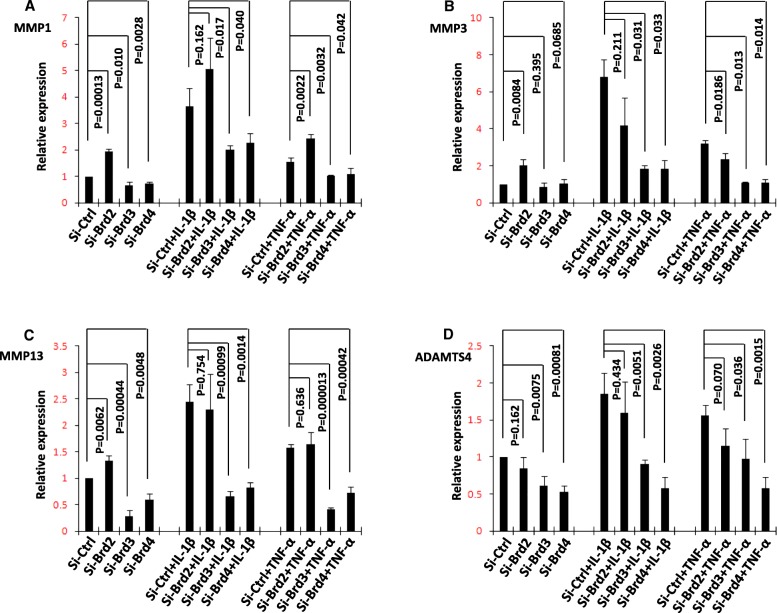
The effect of specific siRNAs on regulation of matrix-degrading enzyme gene transcription in SW1353 cells. SW1353 cells were treated with scrambled siRNA or specific siRNA targeting *BRD2*, *BRD3*, and *BRD4* for 48 h and then treated with or without IL-1β (10 ng/ml) or TNF-α (10 ng/ml) for 6 h. **a**–**d** The transcriptional expression of *MMP1*, *MMP3*, *MMP13*, and *ADAMTS4* was evaluated by real time-PCR. Relative fold-change values were calculated in comparison with the vehicle control that was set to 1 (*n* = 3)

We first verified that transcriptional expression of *MMP1*, *MMP3*, *MMP13*, and *ADAMTS4* was upregulated by IL-1β or TNF-α and repressed by I-BET151 in a human chondrosarcoma cell line (SW1353) (Fig. 1). We then examined the expression of BET proteins by using Western blot assay (Additional file 2: Figure S1) and found the patterns of Brd2, Brd3, and Brd4 protein expression were similar in SW1353 cells and primary human chondrocytes (Fig. 2a). Finally, we performed a knockdown of *BRD2*, *BRD3*, and *BRD4* with corresponding siRNAs in SW1353 cells and confirmed the reduction of Brd2, Brd3, and Brd4 by using Western blot assay (Fig. 2b–d). The analysis of the transcription levels revealed an apparent reduction of inducible transcription of *MMP1*, *MMP3*, *MMP13*, and *ADAMTS4* in the *BRD3* and *BRD4* knockdown cells following stimulation. The reduction of IL-1β-induced *MMP3* transcription, TNF-α-induced *MMP3* transcription, and TNF-α-induced *ADAMTS4* by *BRD3* knockdown and the reduction of IL-1β-induced *MMP3* transcription by *BRD4* knockdown did not reach the criteria of significance, but all the comparisons showed the same trends. *BRD2* knockdown resulted in increased levels of basal and inducible transcription of *MMP1*, *MMP3*, and *MMP13* but not of *ADAMTS4* (Fig. 3a–d).

### IL-1β- or TNF-α-induced recruitment of Brd3 and Brd4 to chromatin

**Fig. 4:**
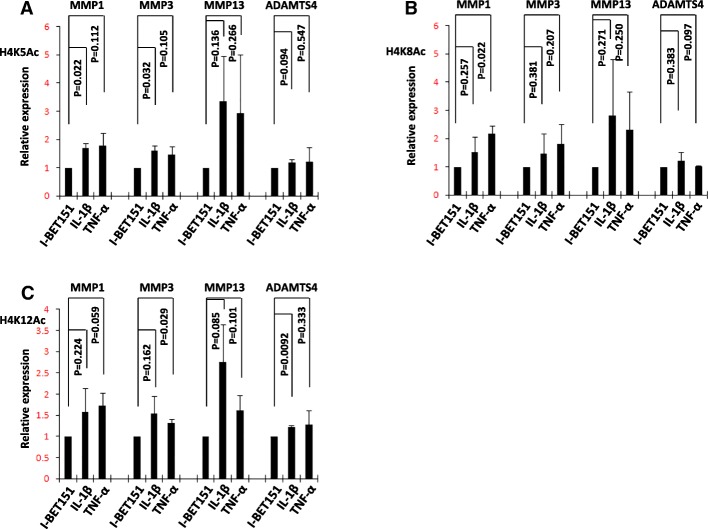
The recruitment of Brd3 and Brd4 to the chromatin responsible for IL-1β- or TNF-α-induced transcription. **a**–**d** Human chondrocytes were pretreated with or without I-BET151 (1 μM), followed by addition of vehicle, IL-1β (10 ng/ml) or TNF-α (10 ng/ml) for 6 h, and ChIP assays were performed for Brd3. **e**–**h** Human chondrocytes were pretreated with or without I-BET151 (1 μM), followed by addition of vehicle, IL-1β (10 ng/ml) or TNF-α (10 ng/ml) for 6 h, and ChIP assays were performed for Brd4. The quantitative analysis of targeted promoter regions was determined by real-time PCR using specific primers for *MMP1*, *MMP3*, *MMP13*, and *ADAMTS4.* Relative fold-change values were calculated in comparison with the vehicle control that was set to 1 (*n* = 2)

We evaluated the recruitment of Brd3 and Brd4 to the promoter regions of *MMP1*, *MMP3*, *MMP13*, and *ADAMTS4* by ChIP assay and found enhanced recruitment of Brd3 and Brd4 to the promoter regions of all the four genes after treatment of IL-1β or TNF-α. The average fold changes were all larger than 2.0, except the recruitment of Brd3 to *MMP3* after the treatment of IL-1β and the recruitment of Brd3 to *MMP3* and Brd4 to *ADAMTS4* after the treatment of TNF-α. I-BET151 abrogated the enhanced recruitment of Brd3 and Brd4 to the promoter regions induced by IL-1β or TNF-α, and significance was found in the reduction of IL-1β-induced recruitment of Brd3 to *MMP13* and TNF-α-induced recruitment of Brd3 to *MMP1*, Brd3 to *MMP13*, Brd4 to *MMP1*, and Brd4 to *MMP13*. But I-BET151 did not show consistent effects on the basal recruitment of Brd3 and Brd4 to the promoter regions of these genes (Fig. 4a–h).

**Fig. 5:**
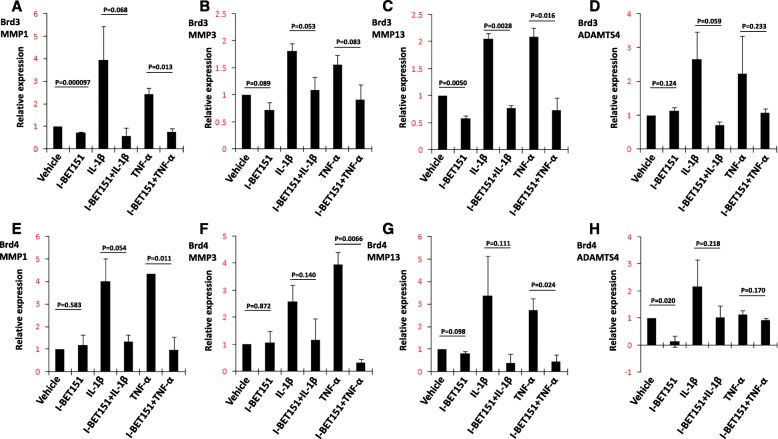
The recruitment of Cdk9 to the chromatin and Ser2 phosphorylation of RNAP II CTD responsible for IL-1β- or TNF-α-induced transcription. **a**–**d** Human chondrocytes were pretreated with or without I-BET151 (1 μM), followed by addition of vehicle, IL-1β (10 ng/ml) or TNF-α (10 ng/ml) for 6 h, and ChIP assays were performed for Cdk9. **e**–**h** Human chondrocytes were pretreated with or without I-BET151 (1 μM), followed by addition of vehicle, IL-1β (10 ng/ml) or TNF-α (10 ng/ml) for 6 h, and ChIP assays were performed for S2P Pol II. The quantitative analysis of targeted promoter regions was determined by real-time PCR using specific primers for *MMP1*, *MMP3*, *MMP13*, and *ADAMTS4.* Relative fold-change values were calculated in comparison with the vehicle control that was set to 1 (*n* = 2)

We also evaluated the recruitment of CDK9 and the Ser2 phosphorylation of RNAP II CTD at the promoter regions of these genes. In parallel with the recruitment of Brd3 and Brd4 to the promoter regions of these genes, we found that IL-1β or TNF-α stimulation led to increased recruitment of CDK9 and Ser2 phosphorylation of RNAP II CTD in all the four genes (Fig. 5a–h). The average fold changes were all larger than 2.0. I-BET151 abrogated both the recruitment of CDK9 and the Ser2 phosphorylation of RNAP II CTD induced by IL-1β or TNF-α, and significance was found in the reduction of TNF-α-induced recruitment of CDK9 to MMP1, IL-1β-induced recruitment of CDK9 to *ADAMTS4*, TNF-α-induced Ser2 phosphorylation of RNAP II CTD in *MMP3*, and IL-1β-induced Ser2 phosphorylation of RNAP II CTD in *MMP1*, *MMP3*, and *MMP13*. I-BET151 did not show consistent effects on the basal recruitment of CDK9 to the promoter regions or basal Ser2 phosphorylation of RNAP II CTD in these genes (Fig. 5a–h).

### IL-1β- or TNF-α-induced modifications of acetylated histone at promoter regions

**Fig. 6:**
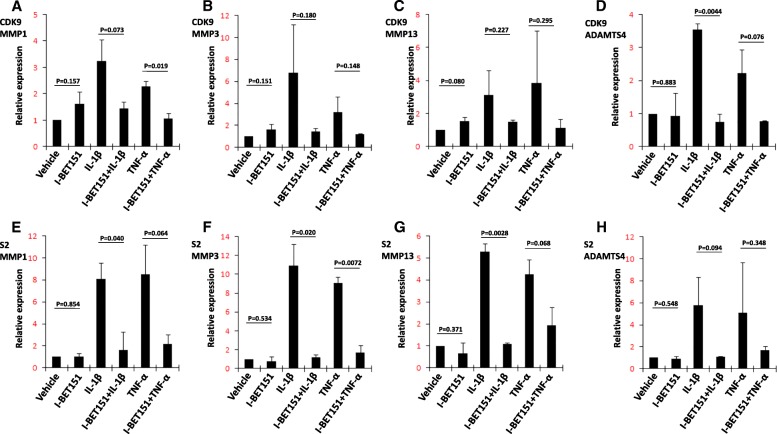
Identification of H4 acetylation after stimulation by IL-1β or TNF-α. **a**–**c** SW1353 cells were stimulated with or without IL-1β (10 ng/ml) or TNF-α (10 ng/ml) for 6 h and analyzed by ChIP for H4K5Ac, H4K8Ac, and H4K12Ac. The quantitative analysis of targeted promoter regions was determined by real-time PCR using specific primers for *MMP1*, *MMP3*, *MMP13*, and *ADAMTS4.* Relative fold-change values were calculated in comparison with the vehicle control that was set to 1 (*n* = 2)

We checked whether IL-1β or TNF-α stimulation could affect these histone modifications at the promoter regions of these inducible genes in chondrocytes, and we found increased levels of these modifications present at *MMP1*, *MMP3*, *MMP13*, and *ADAMTS4* in promoter regions. Significance was found in the IL-1β-induced H4K5Ac at *MMP1*, H4K5Ac at *MMP3*, and H4K12Ac at *ADAMTS4* and in the TNF-α-induced H4K8Ac at *MMP1* and H4K12Ac at *MMP3*. The average fold changes of all the induced modifications present at *MMP1*, *MMP3*, and *MMP13* were larger than 1.5, except TNF-α induced H4K5Ac at *MMP3.* The average fold changes of all the induced modifications present at *ADAMTS4* were between 1 and 1.5(Fig. 6a–c).

## Discussion

In our study, we found that the depletion of Brd4 and Brd3 by siRNA-mediated knockdown resulted in the reduction of IL1β- or TNFα-induced transcription of *MMP1*, *MMP3*, *MMP13*, and *ADAMTS4* in chondrocytes, but depletion of Brd2 did not show such effects. It suggested that both Brd3 and Brd4 were responsible for the IL1β- or TNFα-induced transcription of these genes in chondrocytes.

Increasing amounts of evidence suggest that the recruitment of BET proteins to chromatin is closely associated with their roles in transcriptional regulation [6, 7]. Here, we found enhanced recruitment of Brd3 and Brd4 to the promoter regions of *MMP1*, *MMP3*, *MMP13*, and *ADAMTS4* after the treatment of IL-1β or TNF-α in chondrocytes, and the recruitment could be alleviated by I-BET151. The recruitment of Brd3 and Brd4 is consistent with the expression of these four genes after the treatment of IL-1β or TNF-α in chondrocytes. So, we suspected that the recruitment of Brd3 and Brd4 to the promoter regions was essential for the induction of *MMP1*, *MMP3*, *MMP13*, and *ADAMTS4* by IL-1β or TNF-α in chondrocytes.

There have been studies which reported that Brd4 could directly interact with positive transcription elongation factor b (p-TEFb) [14, 15]. p-TEFb is composed of the CDK9 and cyclin T1 (CycT1) subunits, and CDK9 could phosphorylate Ser2 of the RNA polymerase II C-terminal domain (RNAP II CTD), leading to the release of paused RNAP II during transcriptional elongation [16]. In parallel with enhanced recruitment of Brd3 and Brd4 to the promoter regions of *MMP1*, *MMP3*, *MMP13*, and *ADAMTS4*, we found enhanced recruitment of CDK9 and Ser2 phosphorylation of RNAP II CTD at the promoter regions of all the four genes after the treatment of IL-1β or TNF-α in chondrocytes. When we inhibited the combination of BET proteins and acetylated histones by using I-BET151 in chondrocytes, we found that the IL-1β- or TNF-α-induced recruitment of Brd3, Brd4, and CDK9 to the promoter regions and Ser2 phosphorylation of RNAP II CTD at the promoter regions of these genes were abrogated synchronously. Our results conformed to the previous reports that Brd4 could directly interact with p-TEFb and p-TEFb phosphorylated Ser2 of the RNAP II CTD. The results suggested that the recruitment of Brd4, Brd3, and pTEFb to the chromatin and subsequent Ser2 phosphorylation of RNAP II CTD are correlated with the IL-1β- or TNF-α-induced transcriptional expression of *MMP1*, *MMP3*, *MMP13*, and *ADAMTS4* genes in human chondrocytes.

BET proteins directly interact with several different modifications of acetylated histones, including H4 acetylated at Lys5 (H4K5Ac), H4 acetylated at Lys8 (H4K8Ac), and H4 acetylated at Lys12 (H4K12Ac) [17–19]. Here, we also found increased levels of acetylated H4K5/8/12 and enhanced recruitment of Brd3 and Brd4 to the promoter regions of *MMP1*, *MMP3*, *MMP13*, and *ADAMTS4* after the treatment of IL-1β or TNF-α in chondrocytes. Our results suggested that increased levels of acetylated H4K5/8/12 at promoter regions might be responsible for the enhanced recruitment of Brd3 and Brd4 to the promoter regions of these genes after the treatment of IL-1β or TNF-α in chondrocytes.

Acetylation of histones was reported to be involved in the induction of matrix-degrading enzyme genes by proinflammatory cytokines. There were several reports which suggested NF-κB, JNK, and MAPK pathways were involved in the acetylation of histones [10, 20, 21], and NF-κB, JNK, and MAPK pathways were well known to be involved in the process of activating matrix-degrading enzymes by proinflammatory cytokines [1–3]. TNF could increase the acetylation of histone H3 at the promoter region of another matrix-degrading enzyme, MMP9, and then promote the transcription of *MMP9* [10]. But the inhibitor of HDAC, which could retain the acetylation of histones, was well known to repress the induction of MMPs by proinflammatory cytokines [22–24]. The effect of the inhibitor of HDAC might be mediated by acetylation of α-tubulin, acetylation of NF-κB p65, and so on [23, 25]. So, the effect of the acetylation of histones on the induction of matrix-degrading genes by proinflammatory cytokines is still debatable and complicated, and much of the mechanism remains unknown. Our results suggested that increased levels of acetylated H4K5/8/12 at promoter regions of *MMP1*, *MMP3*, *MMP13*, and *ADAMTS4* genes were associated with enhanced transcriptional expression of these genes in chondrocytes after the treatment of IL-1β or TNF-α.

We found the BET proteins, which acted as “readers” of the histone acetylation, were involved in this process. Brd3 and Brd4 were both shown to be essential for the inducible transcription of *MMP1*, *MMP3*, *MMP13*, and *ADAMTS4* in chondrocytes after the treatment of IL-1β or TNF-α. Enhanced recruitment of Brd3 and Brd4 to the promoter regions could lead to the enhanced recruitment of p-TEFb to the promoter regions and the subsequent Ser2 phosphorylation of RNAP II CTD, and then lead to the enhanced transcription of these genes after the treatment of IL-1β or TNF-α.

Some limitations should be noted in our study. First, because of the limited test times and variety of ChIP assay result, significance was not detected in several comparisons although the trend and fold change were apparent. And we did not perform an electrophoresis assay to get a qualitative ChIP data, which could show visual differences among different treatment groups. Second, we only checked the transcriptional level of the target genes, and a protein level presentation could make the effect of treatments more convincing in this study. And we only used one effective siRNA for each Brd protein to perform the knockdown treatment, although the knockdown effects have been confirmed by Western assay. Third, we suggested the association between increased levels of acetylated H4K5/8/12 and the enhanced recruitment of Brd3 and Brd4 to the promoter regions depending on the previous reports that BET proteins could directly interact with several different modifications of acetylated histones, including acetylated histones H4 [17–19]. But we did not specifically inhibit the acetylation of histones H4 to evaluate the necessity of acetylated histones H4 in this process. Besides, we cannot deny the enhanced acetylation of other histones at the promoter region or any other mechanisms increasing the affinity of BET proteins to the promoter region as the reason for the enhanced recruitment of Brd3 and Brd4 to the promoter regions of these genes in chondrocytes. The exact binding sites of Brd3 and Brd4 were still unknown in our study, and further structural analysis was needed to understand the exact mechanisms.

## Conclusions

**Fig. 7:**
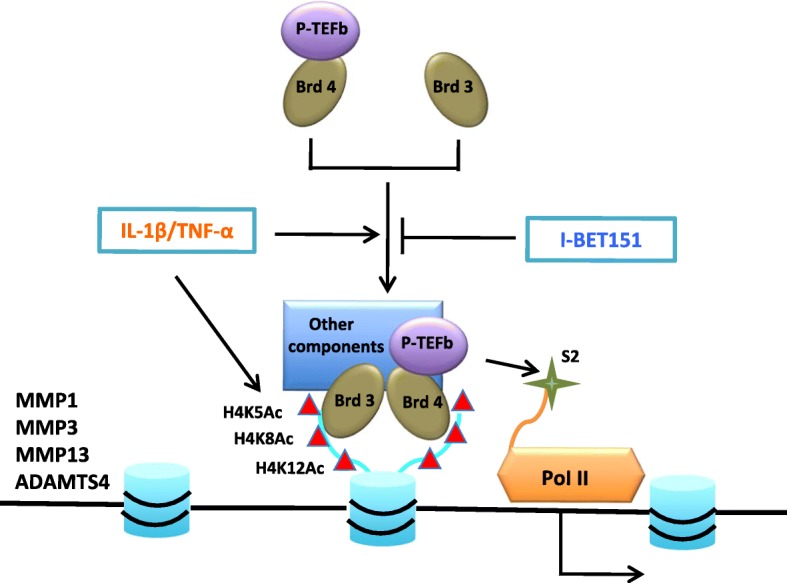
Schematic mechanism for Brd-mediated transcriptional regulation of *MMP1*, *MMP3*, *MMP13*, and *ADAMTS4* genes in human chondrycytes. This model proposes that IL-1β or TNF-α stimulation leads to the H4K5/8/12Ac at TSS regions and the recruitment of Brd3, Brd4, and p-TEFb to these regions. Subsequently, Ser2 RNAP II CTD is phosphorylated by p-TEFb. The recruitment of Brd3 and Brd4 is blocked by I-BET-151

In summary, the results of the present study showed Brd3 and Brd4 were both characterized to be required for IL-1β- or TNF-α-induced transcription of matrix-degrading enzyme genes in chondrocytes. Through recognizing acetylated histone, Brd4 might cooperate with Brd3 to recruit p-TEFb to the chromatin and then phosphorylate the Ser2 of RNAP II CTD in these induced genes during transcriptional activation (Fig. 7).

## Additional files

Additional file 1: **Table S1.** The list of primers used in this study (DOC 36 kb)

Additional file 2: **Figure S1.** The protein expression of the BET family members and the effect of siRNAs in SW1353 cells (A) The relative densitometry of Fig. 2a (*n* = 1). (B) The relative densitometry of Fig. 2b (*n* = 1). (C) The relative densitometry of Fig. 2c (*n* = 1). (D) The relative densitometry of Fig. 2d (*n* = 1). (PPT 186 kb)

## Abbreviations

ADAMTS: A disintegrin-like and metalloproteinase with thrombospondin type 1 motifs
BET: Bromodomain and extraterminal domain
Brd: Bromodomain-containing protein
Cdk9: Cyclin-dependent kinase 9
CycT1: Cyclin T1
DMSO: Dimethyl sulfoxide
GAPDH: Glyceraldehyde-3-phosphate dehydrogenase
H4K12Ac: H4 acetylated at Lys12
H4K5Ac: H4 acetylated at Lys5
H4K8Ac: H4 acetylated at Lys8
IL: Interleukin
JNK: Jun N-terminal kinase,
MAPK: Mitogen-activated protein kinase
MMP: Matrix metalloproteinase
NF-κB: Nuclear factor-κB
OA: Osteoarthritis
p-TEFb: Positive transcription elongation factor b
RNAP II CTD: RNA polymerase II C-terminal domain
S2P Pol II: Ser2-phosphorylated RNA polymerase II C-terminal domain
TNF: Tumor necrosis factor
