# Supplementary material for: Recruitment of Brd3 and Brd4 to acetylated chromatin is essential for proinflammatory cytokine-induced matrix-degrading enzyme expression
Source: J Orthop Surg Res. 2019 Feb 20;14:59. doi: 10.1186/s13018-019-1091-3 (PMC6381721; doi:10.1186/s13018-019-1091-3)
Supplement: Supplementary file 1 — Table S1. The list of primers used in this study (DOC 36 kb) [file 13018_2019_1091_MOESM1_ESM.doc]

Additional file 1: Table S1. The list of primers used in this study

| Gene | Strand | Primer sequences(5’ to 3’) | Detection |
| --- | --- | --- | --- |
| MMP1 | Forward | CTCTGGAGTAATGTCACACCTCT | RT-PCR |
|  | Reverse | TGTTGGTCCACCTTTCATCTTC |  |
|  | Forward | CTGGAAGGGCAAGGACTCTA | ChiP |
|  | Reverse | GGAAGCTGTGAGACACCACA |  |
| MMP3 | Forward | AGTCTTCCAATCCTACTGTTGCT | RT-PCR |
|  | Reverse | TCCCCGTCACCTCCAATCC |  |
|  | Forward | AAAATGCTCCAGTTTTCTCCTC | ChiP |
|  | Reverse | GCAGGACCATTTCCAAACAT |  |
| MMP13 | Forward | ACTGAGAGGCTCCGAGAAATG | RT-PCR |
|  | Reverse | GAACCCCGCATCTTGGCTT |  |
|  | Forward | TGACTGGGAAGTGGAAACCT | ChiP |
|  | Reverse | CGACAATGAGTCCAGCTCAA |  |
| ADAMTS4 | Forward | GAGGAGGAGATCGTGTTTCCA | RT-PCR |
|  | Reverse | CCAGCTCTAGTAGCAGCGTC |  |
|  | Forward | TCCATTCGGCTGCTAGAGAT | ChiP |
|  | Reverse | GTGTCTGTGGGTCTCCCTGT |  |
