# Supplementary material for: Recruitment of Brd3 and Brd4 to acetylated chromatin is essential for proinflammatory cytokine-induced matrix-degrading enzyme expression
Source: J Orthop Surg Res. 2019 Feb 20;14:59. doi: 10.1186/s13018-019-1091-3 (PMC6381721; doi:10.1186/s13018-019-1091-3)
Supplement: Supplementary file 2 — Figure S1. The protein expression of the BET family members and the effect of siRNAs in SW1353 cells (A) The relative densitometry of Fig. 2a (n = 1). (B) The relative densitometry of Fig. 2b (n = 1). (C) The relative densitometry of Fig. 2c (n = 1). (D) The relative densitometry of Fig. 2d (n = 1). (PPT 186 kb) [file 13018_2019_1091_MOESM2_ESM.ppt]

## Slide 1
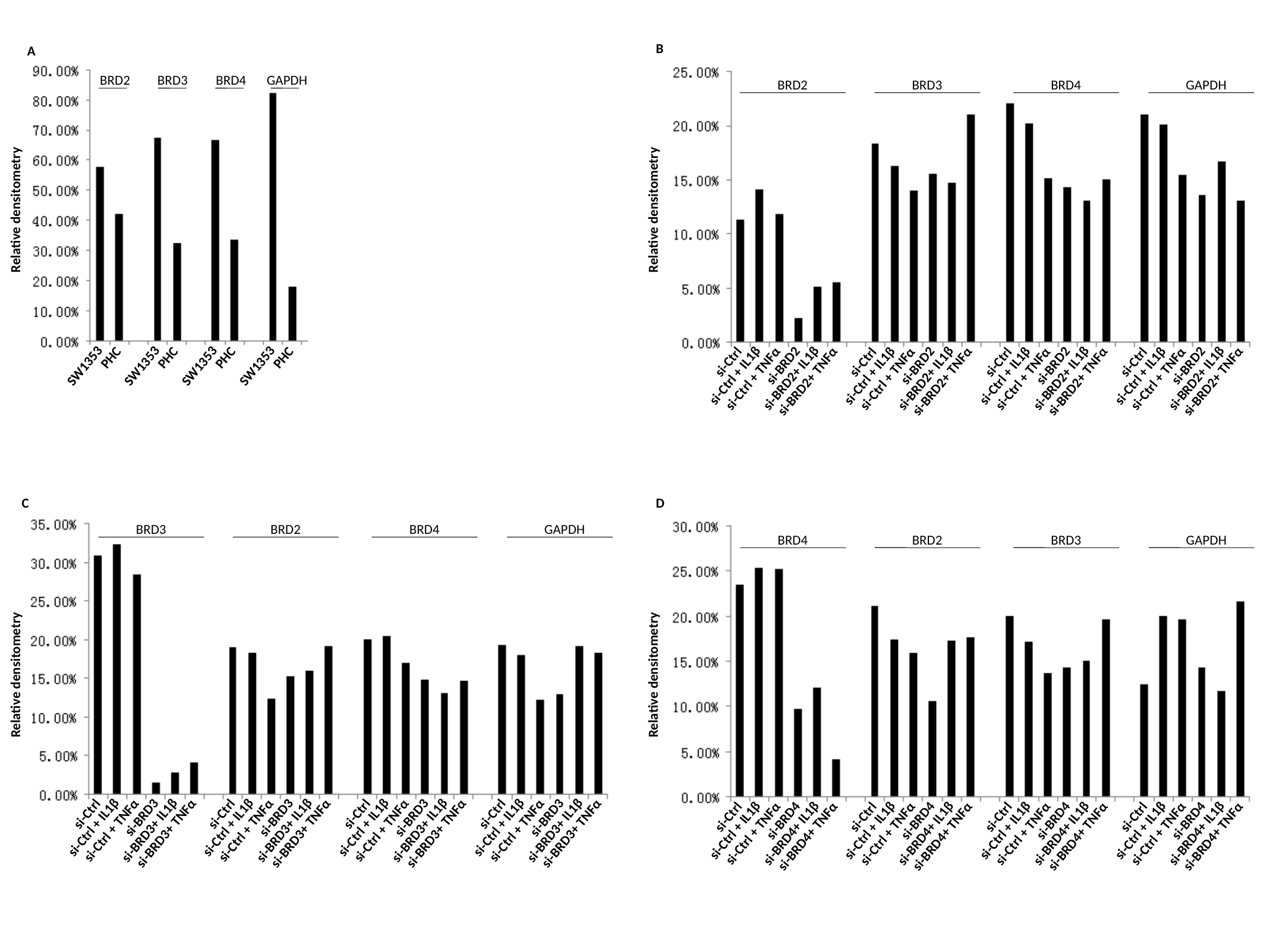

A
B
BRD3
BRD4
GAPDH
BRD2
SW1353
PHC
SW1353
PHC
SW1353
PHC
SW1353
PHC
BRD3
BRD4
GAPDH
BRD2
si-Ctrl
si-BRD2
si-Ctrl + IL1β
si-BRD2+ IL1β
si-BRD2+ TNFα
si-Ctrl + TNFα
si-Ctrl
si-BRD2
si-Ctrl + IL1β
si-BRD2+ IL1β
si-BRD2+ TNFα
si-Ctrl + TNFα
si-Ctrl
si-BRD2
si-Ctrl + IL1β
si-BRD2+ IL1β
si-BRD2+ TNFα
si-Ctrl + TNFα
si-Ctrl
si-BRD2
si-Ctrl + IL1β
si-BRD2+ IL1β
si-BRD2+ TNFα
si-Ctrl + TNFα
Relative densitometry
Relative densitometry
C
D
BRD2
BRD4
GAPDH
BRD3
si-Ctrl
si-BRD3
si-Ctrl + IL1β
si-BRD3+ IL1β
si-BRD3+ TNFα
si-Ctrl + TNFα
si-Ctrl
si-BRD3
si-Ctrl + IL1β
si-BRD3+ IL1β
si-BRD3+ TNFα
si-Ctrl + TNFα
si-Ctrl
si-BRD3
si-Ctrl + IL1β
si-BRD3+ IL1β
si-BRD3+ TNFα
si-Ctrl + TNFα
si-Ctrl
si-BRD3
si-Ctrl + IL1β
si-BRD3+ IL1β
si-BRD3+ TNFα
si-Ctrl + TNFα
BRD2
BRD3
GAPDH
BRD4
si-Ctrl
si-BRD4
si-Ctrl + IL1β
si-BRD4+ IL1β
si-BRD4+ TNFα
si-Ctrl + TNFα
si-Ctrl
si-BRD4
si-Ctrl + IL1β
si-BRD4+ IL1β
si-BRD4+ TNFα
si-Ctrl + TNFα
si-Ctrl
si-BRD4
si-Ctrl + IL1β
si-BRD4+ IL1β
si-BRD4+ TNFα
si-Ctrl + TNFα
si-Ctrl
si-BRD4
si-Ctrl + IL1β
si-BRD4+ IL1β
si-BRD4+ TNFα
si-Ctrl + TNFα
Relative densitometry
Relative densitometry
